# Comparison of Alginate Mixtures as Wall Materials of *Schizochytrium* Oil Microcapsules Formed by Coaxial Electrospray

**DOI:** 10.3390/polym15122756

**Published:** 2023-06-20

**Authors:** Alejandra Arevalo-Gallegos, Sara P. Cuellar-Bermudez, Elda M. Melchor-Martinez, Hafiz M. N. Iqbal, Roberto Parra-Saldivar

**Affiliations:** 1 School of Engineering and Sciences, Tecnologico de Monterrey, Av. Eugenio Garza Sada 2501, Monterrey 64849, N.L., Mexicoelda.melchor@tec.mx (E.M.M.-M.); hafiz.iqbal@tec.mx (H.M.N.I.); 2 Institute of Advanced Materials for Sustainable Manufacturing, Tecnologico de Monterrey, Av. Eugenio Garza Sada 2501, Monterrey 64849, N.L., Mexico

**Keywords:** encapsulation, alginate, maltodextrin, beta-glucans, DHA

## Abstract

This work evaluated maltodextrin/alginate and β-glucan/alginate mixtures in the food industry as wall materials for the microencapsulation of *Schizochytrium* sp. oil, an important source of the omega-3 fatty acid DHA (docosahexaenoic acid). Results showed that both mixtures display a shear-thinning behavior, although the viscosity is higher in β-glucan/alginate mixtures than in maltodextrin/alginate. Scanning electron microscopy was used to assess the morphology of the microcapsules, which appeared more homogeneous for maltodextrin/alginate. In addition, oil-encapsulation efficiency was higher in maltodextrin/alginate mixtures (90%) than in β-glucan/alginate mixtures (80%). Finally, evaluating the microcapsules’ stability by FTIR when exposed to high temperature (80 °C) showed that maltodextrin/alginate microcapsules were not degraded contrary to the β-glucan/alginate microcapsules. Thus, although high oil-encapsulation efficiency was obtained with both mixtures, the microcapsules’ morphology and prolonged stability suggest that maltodextrin/alginate is a suitable wall material for microencapsulation of *Schizochytrium* sp. oil.

## 1. Introduction

Docosahexaenoic acid (DHA) is an omega-3 polyunsaturated fatty acid essential for human health. DHA is the predominant fatty acid in the membrane phospholipids of the brain gray matter and is also present in the eye retina [1]. DHA is obtained through our diet, and its deficiency has been related to cognitive decline and neurodegenerative diseases such as Alzheimer’s [2]. On the other hand, the beneficial effects of DHA on problems associated with cardiovascular disease, hypertension, and inflammation are well documented [3]. Fish is considered the primary source of omega-3 fatty acids for commercial production. However, there are concerns about its consumption since the overexploitation of marine resources and contamination of oil with heavy metals represent environmental and health concerns that limit consumer acceptance [4]. Fish obtain these omega-3 fatty acids from microalgae, which are primary producers in the marine food chain. Therefore, microalgae cultivation has been proposed to produce DHA to avoid environmental and health problems related to fish consumption. 

Microalgae are microorganisms found in different environments, such as soil and fresh and marine water. They can accumulate from 10 to 60% lipids dry weight [5]. In addition, they can perform autotrophic, mixotrophic, and heterotrophic metabolism. Microalgae species producers of DHA include *Schizochytrium*, *Crypthecodinium*, *Isochrysis*, *Amphidinium*, *Paragymnodinium*, *Prorocentrum*, and *Nannochloropsis* [1,6]. Among these, *Schizochytrium* and *Crypthecodinium* are the primary sources for the production of DHA used in aquaculture, nutritional supplements, and infant formulas [1]. *Schizochytrium* and *Crypthecodinium* are grown heterotrophically in fermenters using organic carbons such as glucose. Lipid content in *Schizochytrium* is about 40% dry weight and 50% dry weight in *Crypthecodinium* [7]. DHA content can reach up to 40% of total lipids in *Schizochytrium* and 60% in *Crypthecodinium* [8]. Nevertheless, *Schizochytrium* has faster growth rates and a weaker cell wall than *Crypthecodinium*, which facilitates oil extraction [8].

Polyunsaturated fatty acids (PUFAs) are very susceptible to oxidation during food preparation and storage. Hydroperoxides are highly unstable and are the first molecules to be released during lipid oxidation, which break apart to generate alcohols, aldehydes, and free fatty acids. These compounds cause rancidness, causing off flavors and smell [9]. Lipid oxidation limits the applications of lipids in foods and is a crucial parameter for quality monitoring. Encapsulation, including microencapsulation, is an innovative technique widely used in the food industry to protect unstable ingredients from degradation by exposure to high temperatures, pH changes, exposure to oxygen, or to disguise unwanted colors or flavors [10]. Microencapsulation has been shown to protect oil from oxidation. For instance, Gulzar et al. [11] reported that shrimp oil microcapsules prepared with kidney bean protein isolate and κ-carrageenan had higher protection against oxidative deterioration than non-encapsulated oil. Similarly, Charles et al. [12] reported that microencapsulated tuna fish oil with starch, maltodextrin, and whey protein had higher oxidative stability. These improved properties can be linked to the wall materials’ effectiveness in minimizing the oil’s contact with oxidizing promoters [13]. Microencapsulating materials can also provide temperature resistance and, thus, increase the stability of the oil [14]. In addition, they can mask the characteristic taste and odor of oil [15], expanding the application of oil microcapsules into various food products. 

The most common technique used to encapsulate oil is spray drying. However, this technique’s main disadvantage comes with the high drying temperatures, which contribute to the oxidation of the oil, both encapsulated and remaining on the surface of the powder [16]. Electrohydrodynamic techniques, including electrospinning and electrospraying, are fast and efficient encapsulation techniques with very high encapsulation efficiencies and no use of heat in the process [17]. Coaxial electrospray, a modification of the original electrospray configuration, describes a process in which two liquids are injected separately into the nozzle using two concentric needles. This configuration has yielded capsules with maximum ingredient retention. For instance, edible oils have been successfully encapsulated with coaxial electrosprays, such as arachidonic acid [18], α-linolenic acid [19], and peppermint oil [20]. However, to the best of the authors’ knowledge, this has not been evaluated for microalgal oil. 

The choice of encapsulation materials is critical to the technique’s success because these materials influence the encapsulation efficiency and the product’s shelf life [21]. Polysaccharides are the most commonly used encapsulating material in the food industry [22]. Alginate is an anionic seaweed-derived polysaccharide composed of D-mannuronate and L-guluronate that forms hydrogels when the negatively charged uronic acid groups interact with cations such as calcium (Ca^2+^) via ionic complexation [23]. Alginate has been used extensively to microencapsulate a variety of materials, such as phycocyanin [24] and gallic acid [25]. However, the encapsulation efficiency of oils using alginate can be low, possibly due to low interaction between the wall and core material or to the porosity that can cause oil leakage [26,27]. However, alginate mixed with other polymers can enhance encapsulation efficiency [27,28]. Maltodextrin is another important polysaccharide used for encapsulation. Maltodextrin is made of vegetable starch and is composed of molecules of β-D-glucose joined together with glycosidic bonds. This polysaccharide is commonly used as an encapsulation material due to its positive effect on the stability of emulsions. It is highly soluble in water, has a neutral flavor, and, most importantly, reduces oxygen permeability, protecting the core material from oxidation [29]. The use of maltodextrin as encapsulating material for oil has been widely reported, for instance, for fish oil, rosemary oil, and essential oils [30,31]. 

Encapsulating materials act as a wall to protect the core material from environmental factors that can induce a compound’s degradation, such as oxygen or temperature exposure. However, encapsulating materials could also increase the health benefits of a product. For instance, β-glucans are known to regulate glucose and insulin levels in people with diabetes, and their immunostimulant and proliferative activity have also been reported [32,33]. Although the use of β-glucans for the encapsulation of hydrophobic compounds such as fish oil [34], resveratrol [35], and sea buckthorn oil [36] has been reported, compared to maltodextrin, β-glucans as encapsulating material have been less studied. Therefore, this study evaluated alginate mixtures with maltodextrins and yeast-derived β-glucans as *Schizochytrium* sp. oil microencapsulating agents. In addition, the encapsulation efficiency, morphology, and the material’s ability to protect the oil from oxidation at high temperatures were also studied. 

## 2. Materials and Methods

### 2.1. Materials

Hexane and maltodextrin (dextrose equivalent 4–7) were purchased from DEQ (Monterrey, Mexico). β-glucans derived from yeast were purchased from Nutriproteomics (Colima, México). *Schizochytrium* sp. oil was kindly provided by BIOMEX (Guadalajara, México). Sodium alginate (W201502), Tween 80, FAME mix, and calcium chloride (CaCl_2_) were purchased from Sigma Aldrich (Toluca, Mexico). Nitrogen was purchased from Grupo Infra (Monterrey, Mexico). 

### 2.2. Electrospraying

Two different solutions of maltodextrin and β-glucans were prepared in a total volume of 100 mL; the concentration of alginate was set at 4% *w*/*v* based on preliminary experiments (data not shown) as well as results published by Yan et al. [24]. (Table 1). Alginate was dissolved at 80 °C under constant agitation with a magnetic stirrer, then cooled to 60 °C to add the other materials (maltodextrin or β-glucan). After these materials were dissolved, agitation was maintained for an extra 20 min at room temperature. The solutions were stored at 4 °C in the dark until further used. Tween 80 was added to all the solutions as an emulsifier (0.20 g per 100 mL solution). Based on previous reports, the amount of oil designated for each experiment was set at 5% (*w*/*v*) [37]. The rheology of the solutions was measured using a Physica MCR 102 rheometer (Anton Paar, Graz, Austria) using a cone plate (CP50) of 49.974 mm diameter with an angle of 0.993° and a gap distance between the geometry and the plate set to 0.101 mm. The calculating constants were: Csr (controlled shear rate) 6 min/s, Css (controlled shear stress) 30.54 Pa/mNm, and motor correction factor 1. The analysis time was 6 s, during which 50 data points were taken per sample. Data were collected using Rheoplus 32 Version 3.40. 

**Figure 1:**
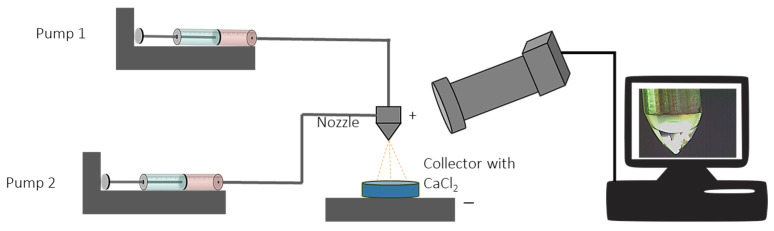
Setup for the coaxial electrospray. Solutions were pumped using pump one and oil pump two. The nozzle was placed 5 cm above the collector containing the 2% *w*/*v* CaCl_2_ solution.

The microcapsules were generated without dilution using a Startup Electrospinning Machine from Yflow (Malaga, Spain) equipped with a variable high voltage 0–30 kV power supply. The setup for the electrospray is shown in Figure 1. As shown, two needles, one containing the oil and another the solutions, were connected to a pump. The oil (core material) was pumped (pump 1) using a syringe of 5 mL (0.6 mm internal diameter needle). The solutions (wall materials) were pumped (pump 2) using a 10 mL syringe (1.4 mm internal diameter needle). The nozzle was placed 5 cm above the collector. A 2% *w*/*v* CaCl_2_ solution was placed in the collector, forming the cross-linked microcapsules. The process was monitored via a CMOS USB Color Camera (Yflow, Malaga, Spain) connected to a computer to visualize the Taylor Cone. The flow rates and voltages used for every sample are presented in Table 2. These voltages were defined based on preliminary experiments to ensure a constant dropping of the different solutions and formation of the Taylor Cone. The capsules were left in the CaCl_2_ solution for 20 min under continuous agitation [38]. The microcapsules were harvested using a self-made aluminum foil plate with a few holes made with a pin. Subsequently, the microcapsules were rinsed with distilled water, allowed to dry at room temperature for 1 h, and stored at 4 °C in the dark until further used. 

### 2.3. Encapsulation Efficiency

Encapsulation efficiency was calculated following Equation (1) [24,39].

(1) $$\%\;\mathrm{Encapsulation}\;\mathrm{efficiency}=\mathrm{initial}\;\mathrm{oil}-\frac{\mathrm{non}\;\mathrm{encapsulated}\;\mathrm{oil}}{\mathrm{initial}\;\mathrm{oil}}\times \;100$$

A sample of the algal oil was analyzed to estimate the total amount of the following fatty acids: palmitoleic acid (C16:1), heptadecenoic acid (C17:0), *cis*-10-heptadecenoic acid (C17:1), γ-linoleic acid (C18:3n6), *cis*-11,14,17-eicosatrienoic acid (C20:3n3), *cis*-4,7,10,13,16,19-docosahexaenoic acid (C22:6n3), and nervonic acid (C24:1n9). The summary of the concentration of these seven fatty acids was taken as the initial oil to encapsulate. The protocol García-Moreno et al. reported [40] to determine non-encapsulated oil was followed with modifications. In short, microcapsules were dissolved in hexane to estimate the concentration of oil encapsulated; the mixture was agitated using a vortex for 1 min and then centrifuged at 4000 rpm for 10 min at 4 °C. Afterward, the aqueous phase was collected and filtered (0.45 μM). A multi-standard FAME mix (fatty acid methyl esters, 10 mg/mL) was added to the samples for fatty acid identification, and undecanoic fatty acid as the internal standard (500 mg/L). The samples were heated to 80 °C for 1 h for transesterification. Once they reached room temperature, 5 mL of hexane was added to each sample. The upper phase was transferred for gas chromatography analysis using a flame ionization detector (GC/FID 6890 Series Agilent Technologies, Santa Clara, CA, USA) equipped with an SP 2380 Column (30 m, 0.25 mm diameter, 0.20 μm of film). The temperature ramp in the oven started at 50 °C for 1 min, followed by an increase up to 240 °C at a rate of 4 °C/min and a holding period of 4 min. The injector was operated at 260 °C and the detector at 280 °C; nitrogen was used as a carrier gas. Once the seven PUFAs were quantified and summed for each sample, the amount of encapsulated oil was determined. The difference between this value and the initial oil was considered non-encapsulated. 

### 2.4. Morphology

Scanning electron microscopy (SEM) analysis was conducted with a Zeiss EVO MA 25 microscope (Zeiss, Oberkochen, Germany) using an acceleration voltage of 20 kV and an EBSD (electron backscatter diffraction) detector. Before taking the images, microcapsules were glazed with gold using a sputter coater under an Ar atmosphere (50 Pa) at 50 mA for 50 s. 

### 2.5. Accelerated Stability

In order to evaluate the effectivity of the encapsulation materials before oxidation caused by high temperatures, an accelerated oxidation test was carried out based on the protocol reported by Gómez-Mascaraque and López-Rubio [41]. Samples of each microcapsule type were placed in an oven at 80 °C, and a sample of the *Schizochytrium* sp. oil was used as a control. After selected time intervals, a sample was taken, and its FTIR spectra were measured using a Perkin Elmer Spectrum 400 FTIR–ATR/NIR instrument (PerkinElmer Scientific, CDMX, Mexico). All the measurements were performed with a germanium crystal ATR objective and recorded in the 4000–450 cm^−1^ spectral range. The samples were dropped in the ATR crystal and measured directly. The cleanliness of the ATR crystal was guaranteed by cleaning it with ethanol in between measurements. Data acquisition and instrument control were accomplished using the Spectrum 10 Spectroscopy Software standard version. 

## 3. Results and Discussion

### 3.1. Formation of Microcapsules

**Figure 2:**
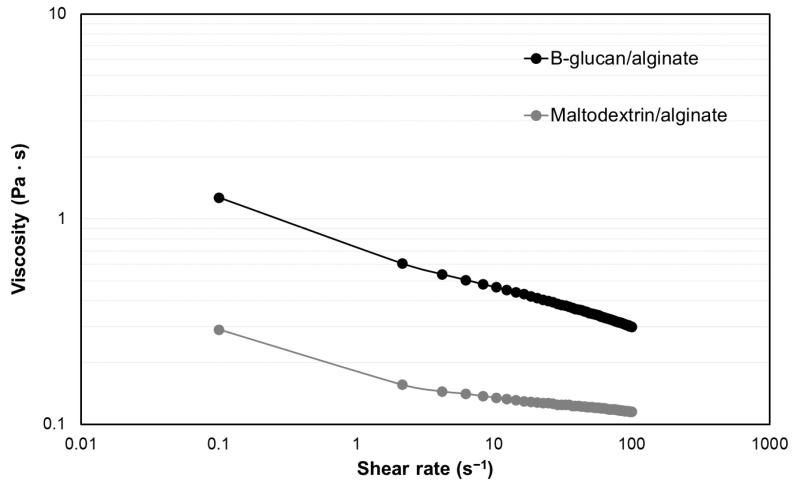
Changes in viscosity at different shear rates of maltodextrin/alginate and β-glucan/alginate solutions. Data represent the average of three replicates.

The viscosity of the solution pumped through the nozzle directly influences the cone and spray formation. The flow curves in Figure 2 show that the two solutions display a shear-thinning behavior. However, the β-glucan/alginate solution had a higher viscosity than the maltodextrin/alginate solution. Maltodextrin shows low viscosity at a high solids concentration [26]. This is an important characteristic of wall materials to evaluate since solutions with a high viscosity require more energy that allows driving the solutions from the meniscus [42]. 

**Figure 3:**
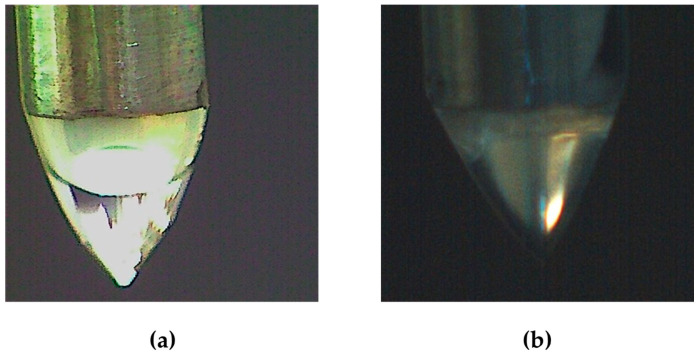
Visualization of the Taylor Cone during the microencapsulation of *Schizochytrium* sp. oil via coaxial electrospray of mixtures of (**a**) maltodextrin/alginate solution and (**b**) β-Glucan/alginate solutions.

Coaxial electrospray uses a coaxial capillary needle to transfer two different fluids independently. The different electrospray modes observed, such as dripping, single-cone, and multiple-cone, are determined by the interaction between the electric stress imposed on the solution and the surface tension stress generated on the liquid–gas interface as the liquid leaves the nozzle [43]. We observed that the maltodextrin/alginate and the β-glucan/alginate solutions could be stabilized in the form of the Taylor Cone (Figure 3). 

Microencapsulation of active substances into polymeric matrices provides a controlled release of the compound since the encapsulating material can act as a carrier for the active compound into the target and protection against thermal or oxidative degradation. The encapsulation efficiency reflects the protective or retaining activity of the active compound by the encapsulating material. High oil-encapsulation efficiency was detected when maltodextrin/alginate was used in this case. Specifically, the encapsulation efficiency of maltodextrin/alginate reached 90%. In the case of the β-glucan/alginate mixture, the encapsulation efficiency was 80%. To date, few studies have evaluated coaxial electrospray for oil encapsulation. For instance, Hu et al. [18] reported 78% encapsulation efficiency for arachidonic acid using zein, while Gómez-Mascaraque et al. [19] reported about 90% of encapsulation efficiency of α-linolenic acid also when using zein as wall material. In another study using alginate/pectin mixtures, Koo et al. [20] reported an 85% encapsulation efficiency for peppermint oil using coaxial electrospray. These studies and our results prove the feasibility and potential of coaxial electrospray. Regarding the matrices used in our study, 68–70% encapsulation efficiencies were reported when juniper berry and sea buckthorn oils were microencapsulated with maltodextrin/gum arabic mixtures [26,36]. In addition, Chen et al. [14] reported a high encapsulation efficiency of 93% for *Schizochytrium* sp. oil in maltodextrin mixtures with soy protein isolate and gum arabic. Similarly, when β-glucan was used as the microencapsulating material of sea buckthorn oil, the encapsulation efficiency was 61–72% [36]. 

**Figure 4:**
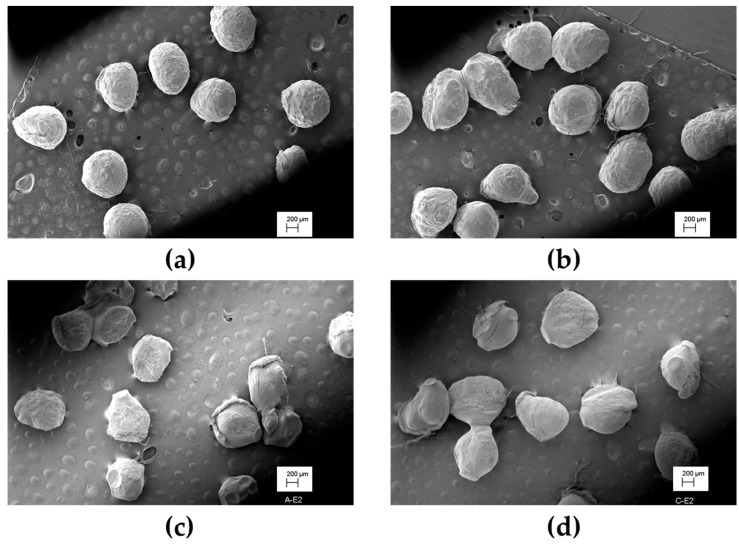
Scanning electron microscopy images of *Schizochytrium* sp. oil microcapsules: (**a**,**b**) maltodextrin/alginate; (**c**,**d**) β-glucan/alginate.

Scanning electron microscopy confirmed microcapsule formation using both solutions (Figure 4). Microcapsules of maltodextrin/alginate appear to have a more defined round shape than those of β-glucan/alginate. These results and a higher encapsulation efficiency suggest that maltodextrin/alginate is preferred for microencapsulating microalgae oil. The stability of the encapsulated oil is discussed in the following section. 

### 3.2. Stability of the Microcapsules

**Figure 5:**
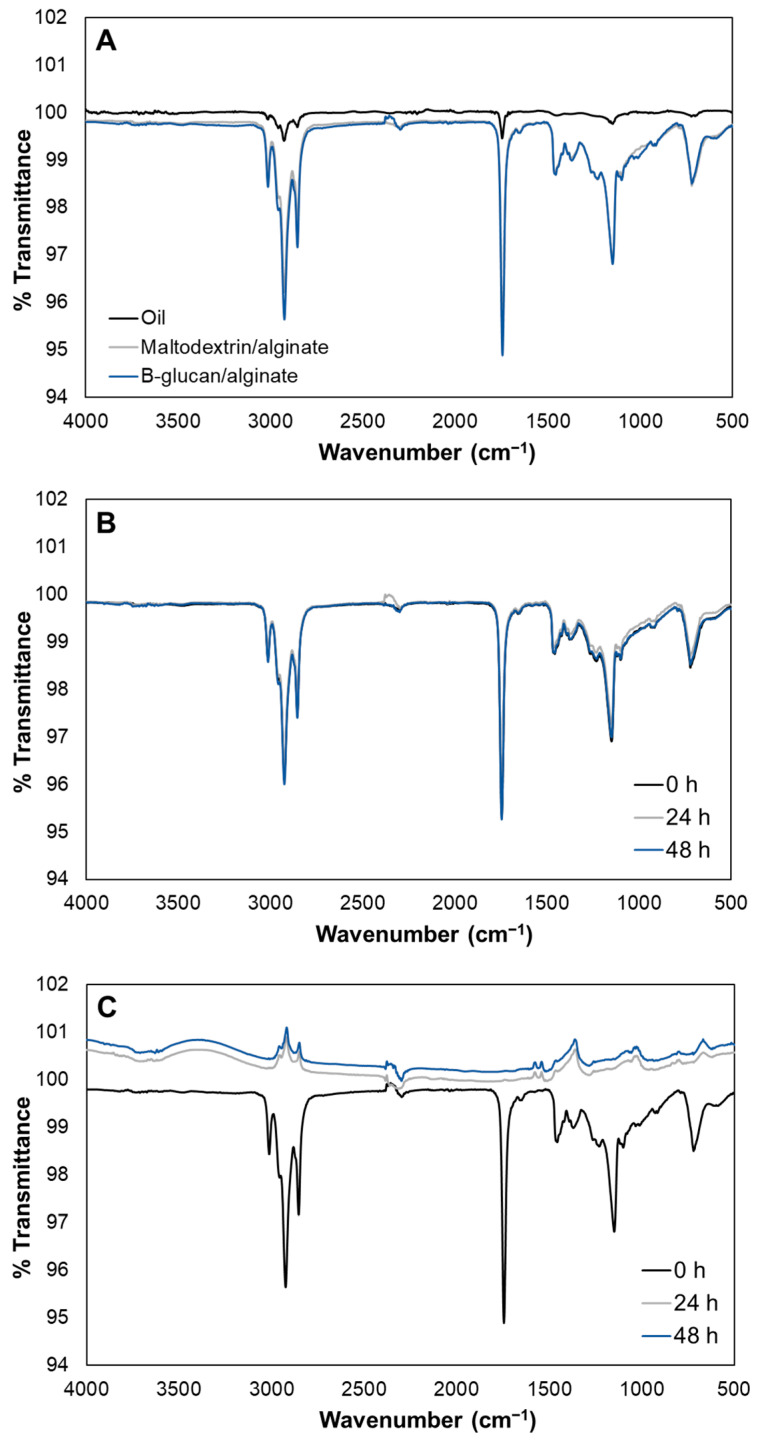
(**A**) FTIR spectra of a sample of oil (non-encapsulated), maltodextrin/alginate oil microcapsules, and β-glucan/alginate oil microcapsules. (**B**) FTIR spectra of maltodextrin/alginate oil microcapsules recorded after exposure at 80 °C. (**C**) FTIR spectra of β-glucan/alginate oil microcapsules recorded after exposure at 80 °C.

FTIR spectrum was used to identify common bands among the oil and microcapsules. Although the oil was encapsulated in a matrix of maltodextrin/alginate or β-glucan/alginate, the spectra of the microcapsules were similar to that of the oil sample. Thus, the identification of the bands was based on previous studies on edible oils (see Lerma-Garcia et al., [44]). Specifically, eight bands were found in common: 3015 cm^−1^, 2960 cm^−1^, 2925 cm^−1^, 2855 cm^−1^, 1745 cm^−1^, 1455 cm^−1^, 1150 cm^−1^, and 725 cm^−1^ (Figure 5A). The band at 3015 cm^−1^ corresponds to =C–H (cis) stretching, and at 2960 cm^−1^ corresponds to –C–H (CH_3_) stretching. The bands at 2925 and 2855 cm^−1^ can be linked to the stretching vibration of methylene (–CH_2_) groups, while the band at 1745 cm^−1^ corresponds to the ester carbonyl (–C=O) functional group. At 1455 cm^−1^, –C–H bending vibrations of CH_2_ and CH_3_ groups can be identified. Stretching of –C–O and bending of –CH_2_– groups correspond to the band at 1150 cm^−1^. At 725 cm^−1^, the bending of –HC=CH– (cis) groups can be identified. The band at 725 cm^−1^ also overlaps with rocking vibrations of –(CH_2_)_n_– groups. Differences in the transmittance intensity can be observed between the oil and the microcapsules (Figure 5A). However, there is no shifting in the frequency of the primary functional groups of the oil, and bands corresponding to oxidation products from oil [45], such as hydroperoxides (3450 cm^−1^) and alcohols (3530 cm^−1^), did not appear.

Microencapsulation aims to protect and prolong the shelf life of a product, in this case, *Schizochytrium* sp. oil. Thus, the stability of the microcapsules was evaluated by considering two factors, temperature and time. The microcapsules were exposed to a temperature increase (80 °C) before recording the FTIR spectra after 24 and 48 h. As shown in Figure 5B, the spectra of the maltodextrin/alginate did not change even after 48 h of exposure, suggesting that the oil is not degraded and can be preserved longer. On the other hand, the spectra of the β-glucan/alginate microcapsules were completely different after 24 h of heat exposure (Figure 5C). The difference in the spectra footprint can be interpreted as the degradation of the microcapsules and oil. Degradation of β-glucan by heat has been previously reported, as reviewed by Mejía et al. [46]. Thus, maltodextrin/alginate mixtures are suitable for preserving *Schizochytrium* sp. oil. Previous studies have also reported the suitable stability of maltodextrin as a wall material. For instance, Pourashouri et al. [16] reported suitable oxidative stability and low peroxide values when fish oil was microencapsulated in maltodextrin mixtures. Similarly, Chen et al. [14] reported low peroxide values for the microencapsulated *Schizochytrium* sp. oil compared to the non-encapsulated oil for up to 8 weeks at 45 °C.

Alginate is a polysaccharide commonly reported as an encapsulating material due to its biocompatibility and non-toxicity [47]. Maltodextrin is an excellent encapsulating material due to its high solubility, low viscosity, neutral taste and aroma, and low cost [31]. Maltodextrin is one of the most used wall materials for the microencapsulation of oils, independently of the technique used, i.e., spray drying or electrospray [48]. Maltodextrin has proven suitable thermal stability that protects oils against oxidation [49]. As recently reviewed by Perez-Palacios et al. [48], several studies confirm the stability of maltodextrin as a wall material. For instance, volatile compounds from omega-3 fatty acid oxidation with rancid and off-flavor perceptions were not detected for microencapsulated fish oil in maltodextrin and maltodextrin/chitosan mixtures. The application of *Schizochytrium* sp. oil microcapsules is diverse since fish oil microcapsules have already been incorporated into bread, ice cream, or chocolate [50]. In addition, these oil microcapsules could be used as fat replacers. Specifically, maltodextrin microcapsules have been used as natural food colorants, antioxidants, ingredients, preservatives, or personal care products [31]. Thus, the combination of alginate and maltodextrin as wall material is promising.

Future work is envisaged, including differential scanning calorimetry to study the thermal properties of the capsules and lipid oxidation. It is also necessary to evaluate the microencapsulates’ digestibility to determine the accessibility to the oil during digestion. Finally, it is envisaged to assess the microcapsules in food formulation to study their behavior in a complex system. 

## 4. Conclusions

Microalgae, including *Schizochytrium* sp., are a key source of essential fatty acids. In this work, we demonstrated the feasibility of using alginate/maltodextrin and β-glucan/alginate as wall materials for the microencapsulation of *Schizochytrium* sp. oil through coaxial electrospray. However, alginate/maltodextrin showed better encapsulation efficiency, morphology, and stability properties than β-glucan/alginate. The microencapsulation of oil offers new opportunities in food technology for oils from microalgae. In addition, these microcapsules allow the nutritional enrichment of products by adding essential fatty acids that could substitute saturated fats. 

## Figures and Tables

**Table 1:** Composition of the encapsulation materials for the electrospray process.

	% Material (*w*/*v*)			% Core Material (*w*/*v*)
Solution	Alginate	Maltodextrin	β-Glucan	*Schizochytrium* sp. Oil
1	4	2	-	5
2	4	-	2	5

**Table 2:** Operational parameters for the production of microcapsules with coaxial electrospray.

Solution	Material	Flow Pump 1 (μL/min)	Flow Pump 2 (μL/min)	Nozzle Voltage (kV)	Collector Voltage (kV)
1	Maltodextrin/alginate	10	4	5.2	−4.4
2	β-glucan/alginate	10	2	4.6	−4.1

## Data Availability

Data will be made available on request.
